# Low-temperature rerefinement of non­merohedrally twinned tripyridinium bis­[tetra­bromidoferrate(III)] bromide

**DOI:** 10.1107/S1600536809007090

**Published:** 2009-03-14

**Authors:** Seik Weng Ng

**Affiliations:** a Department of Chemistry, University of Malaya, 50603 Kuala Lumpur, Malaysia

## Abstract

The asymmetric unit of the title double salt, (C_5_H_6_N)_3_[FeBr_4_]_2_Br, consists of three pyridinium cations, two tetra­hedral bromidoferrate(III) anions and a bromide anion. The three cations each form one N—H⋯Br  hydrogen bond to the bromide anion. The crystal under investigation was a non-merohedral twin, with a portion of 22% for the minor twin component.

## Related literature

The authors of the original room-temperature study noted twinning but the refinement program then could not take this into consideration; see: Lowe *et al.* (1994 ▶).
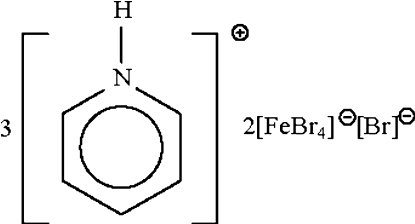


## Experimental

### 

#### Crystal data

(C_5_H_6_N)_3_[FeBr_4_]_2_Br
*M* *_r_* = 1071.21
Monoclinic, 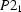

*a* = 7.5602 (1) Å
*b* = 14.0125 (2) Å
*c* = 13.5609 (2) Å
β = 95.172 (1)°
*V* = 1430.76 (3) Å^3^
*Z* = 2
Mo *K*α radiation
μ = 13.59 mm^−1^
*T* = 123 K
0.30 × 0.25 × 0.20 mm

#### Data collection

Bruker SMART APEX diffractometer
Absorption correction: multi-scan (*SADABS*; Sheldrick, 1996 ▶) *T*_min_ = 0.098, *T*_max_ = 0.154
13428 measured reflections
6460 independent reflections
6006 reflections with *I* > 2σ(*I*)
*R*_int_ = 0.038

#### Refinement

*R*[*F* ^2^ > 2σ(*F* ^2^)] = 0.043
*wR*(*F* ^2^) = 0.106
*S* = 1.07
6460 reflections
263 parameters
109 restraints
H-atom parameters constrained
Δρ_max_ = 1.29 e Å^−3^
Δρ_min_ = −1.71 e Å^−3^
Absolute structure: Flack (1983 ▶), 3046 Friedel pairs
Flack parameter: 0.10 (2)

### 

Data collection: *APEX2* (Bruker, 2008 ▶); cell refinement: *SAINT* (Bruker, 2008 ▶); data reduction: *SAINT*; program(s) used to solve structure: *SHELXS97* (Sheldrick, 2008 ▶); program(s) used to refine structure: *SHELXL97* (Sheldrick, 2008 ▶) and *PLATON* (Spek, 2009 ▶); molecular graphics: *X-SEED* (Barbour, 2001 ▶); software used to prepare material for publication: *publCIF* (Westrip, 2009 ▶).

## Supplementary Material

Crystal structure: contains datablocks global, I. DOI: 10.1107/S1600536809007090/tk2380sup1.cif

Structure factors: contains datablocks I. DOI: 10.1107/S1600536809007090/tk2380Isup2.hkl

Additional supplementary materials:  crystallographic information; 3D view; checkCIF report

## Figures and Tables

**Table 1:** Hydrogen-bond geometry (Å, °)

*D*—H⋯*A*	*D*—H	H⋯*A*	*D*⋯*A*	*D*—H⋯*A*
N1—H1⋯Br9	0.88	2.35	3.202 (9)	163
N2—H2⋯Br9	0.88	2.59	3.292 (8)	137
N3—H3⋯Br9	0.88	2.52	3.279 (7)	146

**Fig. 1.**
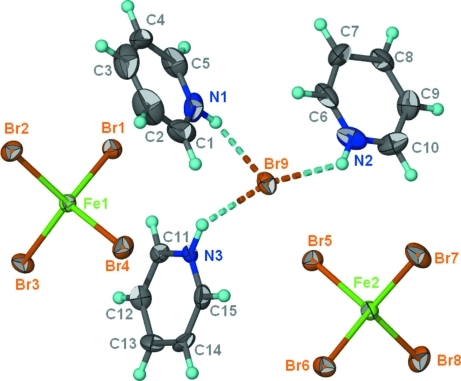
Thermal ellipsoid plot (Barbour, 2001) of [C5H6N]3[FeBr4]2[Br].

## supplementary crystallographic information

### Experimental

The crystals were provided by Dr. Nasser Safari of Shahid Beheshti University. Pyridine (2.2 ml, 25 mmol) was added to a solution of ferric bromide (1.25 g, 4.23 mmol) dissolved in a mixture of 1.2 *M* hydrobromic acid and 2.4 *M* acetic acid (20 ml). The red solution was set aside for two weeks, after which crystals separated out.

### Refinement

The refinement initally converged to an *R*_1_ value of 0.088, but there were large peaks/deep holes. The crystal is in fact a nonmerohedral twin. The law, as given by *PLATON* (Spek, 2003), is (-1 0 0, 0 - 1 0, 0.323 0 1). The refinement, with an approximate twin component of 22%, halved the *R*_1_ index. The twinning affected the anisotropic temperature factors of the carbon and nitrogen atoms; these were restrained to be nearly isotropic.

Carbon- and nitrogen-bound H-atoms were placed in calculated positions (C–H 0.95, N–H 0.88 Å) and were included in the refinement in the riding model approximation, with *U*(H) set to 1.2*U*(C,*N*).

The final difference Fourier map had large peaks/holes in the vicinity of the bromide atoms.

### Crystal data

**Table:**

(C_5_H_6_N)_3_[FeBr_4_]_2_Br	*F*(000) = 992
*M**_r_* = 1071.21	*D*_x_ = 2.487 Mg m^−^^3^
Monoclinic, *P*2_1_	Mo *K*α radiation, λ = 0.71073 Å
Hall symbol: P 2yb	Cell parameters from 8983 reflections
*a* = 7.5602 (1) Å	θ = 2.7–28.3°
*b* = 14.0125 (2) Å	µ = 13.59 mm^−^^1^
*c* = 13.5609 (2) Å	*T* = 123 K
β = 95.172 (1)°	Irregular block, brown
*V* = 1430.76 (3) Å^3^	0.30 × 0.25 × 0.20 mm
*Z* = 2	

### Data collection

**Table:**

Bruker SMART APEX diffractometer	6460 independent reflections
Radiation source: fine-focus sealed tube	6006 reflections with *I* > 2σ(*I*)
graphite	*R*_int_ = 0.038
ω scans	θ_max_ = 27.5°, θ_min_ = 1.5°
Absorption correction: multi-scan (*SADABS*; Sheldrick, 1996)	*h* = −9→9
*T*_min_ = 0.098, *T*_max_ = 0.154	*k* = −18→18
13428 measured reflections	*l* = −17→17

### Refinement

**Table:**

Refinement on *F*^2^	Secondary atom site location: difference Fourier map
Least-squares matrix: full	Hydrogen site location: inferred from neighbouring sites
*R*[*F*^2^ > 2σ(*F*^2^)] = 0.043	H-atom parameters constrained
*wR*(*F*^2^) = 0.106	*w* = 1/[σ^2^(*F*_o_^2^) + (0.0502*P*)^2^ + 4.7139*P*] where *P* = (*F*_o_^2^ + 2*F*_c_^2^)/3
*S* = 1.07	(Δ/σ)_max_ = 0.001
6460 reflections	Δρ_max_ = 1.29 e Å^−^^3^
263 parameters	Δρ_min_ = −1.71 e Å^−^^3^
109 restraints	Absolute structure: Flack (1983), 3046 Friedel pairs
Primary atom site location: structure-invariant direct methods	Flack parameter: 0.10 (2)

### Fractional atomic coordinates and isotropic or equivalent isotropic displacement parameters (Å^2^)

**Table:**

	*x*	*y*	*z*	*U*_iso_*/*U*_eq_	
Br1	0.27784 (12)	0.49992 (6)	0.74070 (6)	0.02428 (19)	
Br2	0.27108 (13)	0.35971 (6)	0.50468 (7)	0.0250 (2)	
Br3	0.02833 (11)	0.60181 (6)	0.50814 (7)	0.02457 (19)	
Br4	0.52742 (11)	0.59765 (7)	0.52624 (7)	0.0268 (2)	
Br5	0.52708 (11)	0.89250 (6)	0.95876 (7)	0.02335 (19)	
Br6	0.75284 (12)	1.00789 (7)	0.74713 (6)	0.02522 (19)	
Br7	1.02585 (12)	0.91096 (8)	0.97548 (8)	0.0356 (3)	
Br8	0.74006 (15)	1.13935 (7)	0.98802 (7)	0.0350 (2)	
Br9	0.74762 (11)	0.65558 (7)	0.83903 (6)	0.02359 (19)	
Fe1	0.27879 (15)	0.51483 (9)	0.56879 (9)	0.0177 (2)	
Fe2	0.76356 (16)	0.98860 (9)	0.91855 (9)	0.0197 (3)	
N1	0.7426 (11)	0.4395 (6)	0.7619 (7)	0.038 (2)	
H1	0.7427	0.4931	0.7959	0.045*	
N2	0.7876 (12)	0.7029 (7)	1.0778 (6)	0.0343 (19)	
H2	0.7993	0.7233	1.0173	0.041*	
N3	0.3634 (10)	0.7457 (5)	0.7543 (5)	0.0203 (15)	
H3	0.4324	0.7032	0.7863	0.024*	
C1	0.8048 (15)	0.4412 (10)	0.6741 (9)	0.047 (3)	
H1A	0.8457	0.4988	0.6470	0.056*	
C2	0.8080 (17)	0.3569 (11)	0.6239 (9)	0.054 (3)	
H2A	0.8582	0.3549	0.5622	0.065*	
C3	0.7408 (16)	0.2758 (9)	0.6606 (10)	0.048 (3)	
H3A	0.7363	0.2182	0.6234	0.058*	
C4	0.6797 (14)	0.2790 (8)	0.7523 (9)	0.038 (2)	
H4	0.6371	0.2226	0.7810	0.046*	
C5	0.6802 (15)	0.3618 (9)	0.8016 (7)	0.038 (2)	
H5	0.6357	0.3647	0.8649	0.046*	
C6	0.7428 (14)	0.6150 (8)	1.0919 (7)	0.035 (2)	
H6	0.7246	0.5738	1.0363	0.042*	
C7	0.7210 (16)	0.5797 (8)	1.1832 (9)	0.041 (3)	
H7	0.6873	0.5151	1.1919	0.049*	
C8	0.7500 (12)	0.6418 (8)	1.2642 (7)	0.032 (2)	
H8	0.7361	0.6199	1.3294	0.039*	
C9	0.7984 (14)	0.7340 (8)	1.2482 (7)	0.035 (2)	
H9	0.8192	0.7771	1.3021	0.042*	
C10	0.8163 (16)	0.7633 (7)	1.1548 (8)	0.039 (2)	
H10	0.8497	0.8274	1.1433	0.047*	
C11	0.1903 (12)	0.7447 (6)	0.7669 (6)	0.0208 (17)	
H11	0.1435	0.6989	0.8092	0.025*	
C12	0.0815 (12)	0.8104 (7)	0.7182 (6)	0.0260 (18)	
H12	−0.0420	0.8102	0.7262	0.031*	
C13	0.1503 (13)	0.8766 (6)	0.6576 (7)	0.0256 (19)	
H13	0.0757	0.9231	0.6241	0.031*	
C14	0.3330 (13)	0.8744 (6)	0.6459 (7)	0.0260 (19)	
H14	0.3831	0.9190	0.6035	0.031*	
C15	0.4375 (12)	0.8082 (7)	0.6956 (7)	0.0269 (19)	
H15	0.5614	0.8062	0.6888	0.032*	

### Atomic displacement parameters (Å^2^)

**Table:**

	*U*^11^	*U*^22^	*U*^33^	*U*^12^	*U*^13^	*U*^23^
Br1	0.0313 (4)	0.0211 (4)	0.0195 (4)	−0.0010 (4)	−0.0025 (3)	−0.0005 (3)
Br2	0.0325 (5)	0.0188 (4)	0.0241 (4)	−0.0009 (4)	0.0039 (4)	−0.0032 (3)
Br3	0.0164 (4)	0.0285 (5)	0.0285 (4)	0.0046 (4)	0.0009 (3)	0.0077 (4)
Br4	0.0168 (4)	0.0268 (5)	0.0369 (5)	−0.0041 (4)	0.0030 (3)	0.0016 (4)
Br5	0.0181 (4)	0.0236 (4)	0.0286 (4)	−0.0015 (3)	0.0038 (3)	0.0022 (3)
Br6	0.0311 (4)	0.0272 (4)	0.0175 (4)	0.0014 (4)	0.0031 (3)	0.0012 (4)
Br7	0.0186 (4)	0.0520 (7)	0.0356 (5)	0.0033 (4)	−0.0007 (4)	0.0137 (5)
Br8	0.0507 (6)	0.0292 (6)	0.0246 (5)	−0.0099 (5)	0.0008 (4)	−0.0090 (4)
Br9	0.0204 (4)	0.0272 (5)	0.0225 (4)	0.0047 (4)	−0.0019 (3)	−0.0033 (3)
Fe1	0.0153 (5)	0.0167 (6)	0.0206 (6)	0.0002 (5)	−0.0005 (4)	−0.0003 (5)
Fe2	0.0186 (5)	0.0230 (6)	0.0173 (6)	−0.0020 (5)	0.0003 (4)	0.0008 (5)
N1	0.025 (4)	0.030 (4)	0.056 (5)	0.003 (3)	−0.013 (4)	−0.012 (4)
N2	0.038 (4)	0.043 (5)	0.023 (4)	0.011 (4)	0.010 (3)	0.009 (3)
N3	0.020 (3)	0.019 (3)	0.021 (3)	−0.002 (3)	−0.006 (3)	0.002 (3)
C1	0.030 (5)	0.055 (6)	0.055 (6)	−0.009 (5)	−0.004 (4)	0.028 (5)
C2	0.042 (6)	0.083 (8)	0.038 (5)	0.003 (6)	0.009 (5)	0.000 (6)
C3	0.040 (5)	0.047 (6)	0.056 (6)	0.008 (5)	−0.005 (5)	−0.021 (5)
C4	0.028 (5)	0.028 (5)	0.055 (6)	−0.007 (4)	−0.013 (4)	0.016 (4)
C5	0.036 (5)	0.058 (6)	0.021 (4)	0.001 (5)	0.005 (4)	−0.002 (4)
C6	0.043 (5)	0.035 (5)	0.024 (4)	0.013 (4)	−0.011 (4)	−0.005 (4)
C7	0.044 (5)	0.026 (5)	0.051 (6)	−0.013 (4)	−0.009 (5)	0.005 (4)
C8	0.028 (4)	0.045 (5)	0.024 (4)	0.000 (4)	−0.001 (3)	0.010 (4)
C9	0.037 (5)	0.039 (5)	0.029 (5)	−0.002 (4)	0.004 (4)	−0.013 (4)
C10	0.049 (5)	0.022 (4)	0.048 (5)	0.002 (4)	0.010 (5)	0.001 (4)
C11	0.023 (4)	0.019 (4)	0.021 (4)	−0.002 (3)	0.004 (3)	−0.001 (3)
C12	0.023 (4)	0.031 (4)	0.025 (4)	0.001 (4)	0.004 (3)	−0.004 (3)
C13	0.027 (4)	0.020 (4)	0.029 (4)	0.008 (4)	0.001 (3)	0.005 (3)
C14	0.028 (4)	0.020 (4)	0.031 (4)	−0.010 (3)	0.008 (4)	0.005 (3)
C15	0.021 (4)	0.032 (4)	0.028 (4)	−0.007 (4)	−0.003 (3)	−0.003 (4)

### Geometric parameters (Å, °)

**Table:**

Br1—Fe1	2.341 (2)	C3—H3A	0.9500
Br2—Fe1	2.340 (2)	C4—C5	1.340 (16)
Br3—Fe1	2.338 (1)	C4—H4	0.9500
Br4—Fe1	2.326 (1)	C5—H5	0.9500
Br5—Fe2	2.342 (1)	C6—C7	1.357 (15)
Br6—Fe2	2.335 (1)	C6—H6	0.9500
Br7—Fe2	2.331 (2)	C7—C8	1.404 (15)
Br8—Fe2	2.326 (2)	C7—H7	0.9500
N1—C1	1.319 (15)	C8—C9	1.366 (15)
N1—C5	1.320 (15)	C8—H8	0.9500
N1—H1	0.8800	C9—C10	1.349 (15)
N2—C6	1.296 (14)	C9—H9	0.9500
N2—C10	1.347 (14)	C10—H10	0.9500
N2—H2	0.8800	C11—C12	1.364 (13)
N3—C11	1.335 (11)	C11—H11	0.9500
N3—C15	1.340 (12)	C12—C13	1.372 (13)
N3—H3	0.8800	C12—H12	0.9500
C1—C2	1.36 (2)	C13—C14	1.405 (13)
C1—H1A	0.9500	C13—H13	0.9500
C2—C3	1.357 (19)	C14—C15	1.358 (13)
C2—H2A	0.9500	C14—H14	0.9500
C3—C4	1.366 (17)	C15—H15	0.9500
			
Br4—Fe1—Br3	107.44 (6)	N1—C5—C4	119.7 (9)
Br4—Fe1—Br2	111.41 (6)	N1—C5—H5	120.2
Br3—Fe1—Br2	111.18 (6)	C4—C5—H5	120.2
Br4—Fe1—Br1	111.51 (6)	N2—C6—C7	122.4 (10)
Br3—Fe1—Br1	108.77 (6)	N2—C6—H6	118.8
Br2—Fe1—Br1	106.55 (6)	C7—C6—H6	118.8
Br8—Fe2—Br7	112.54 (6)	C6—C7—C8	117.7 (10)
Br8—Fe2—Br6	107.52 (6)	C6—C7—H7	121.1
Br7—Fe2—Br6	109.63 (6)	C8—C7—H7	121.1
Br8—Fe2—Br5	109.89 (6)	C9—C8—C7	119.1 (9)
Br7—Fe2—Br5	107.40 (6)	C9—C8—H8	120.4
Br6—Fe2—Br5	109.86 (6)	C7—C8—H8	120.4
C1—N1—C5	123.5 (10)	C10—C9—C8	119.2 (10)
C1—N1—H1	118.2	C10—C9—H9	120.4
C5—N1—H1	118.2	C8—C9—H9	120.4
C6—N2—C10	120.6 (9)	N2—C10—C9	121.0 (10)
C6—N2—H2	119.7	N2—C10—H10	119.5
C10—N2—H2	119.7	C9—C10—H10	119.5
C11—N3—C15	123.4 (8)	N3—C11—C12	119.1 (8)
C11—N3—H3	118.3	N3—C11—H11	120.4
C15—N3—H3	118.3	C12—C11—H11	120.4
N1—C1—C2	117.3 (11)	C11—C12—C13	120.1 (8)
N1—C1—H1A	121.4	C11—C12—H12	120.0
C2—C1—H1A	121.4	C13—C12—H12	120.0
C3—C2—C1	121.2 (11)	C12—C13—C14	118.9 (8)
C3—C2—H2A	119.4	C12—C13—H13	120.6
C1—C2—H2A	119.4	C14—C13—H13	120.6
C2—C3—C4	118.3 (11)	C15—C14—C13	119.5 (8)
C2—C3—H3A	120.8	C15—C14—H14	120.3
C4—C3—H3A	120.8	C13—C14—H14	120.3
C5—C4—C3	119.8 (10)	N3—C15—C14	119.1 (8)
C5—C4—H4	120.1	N3—C15—H15	120.5
C3—C4—H4	120.1	C14—C15—H15	120.5
			
C5—N1—C1—C2	−1.8 (17)	C7—C8—C9—C10	0.3 (17)
N1—C1—C2—C3	3.5 (19)	C6—N2—C10—C9	−0.3 (17)
C1—C2—C3—C4	−4.1 (19)	C8—C9—C10—N2	−0.2 (17)
C2—C3—C4—C5	2.9 (18)	C15—N3—C11—C12	−0.2 (13)
C1—N1—C5—C4	0.6 (17)	N3—C11—C12—C13	−0.3 (13)
C3—C4—C5—N1	−1.2 (17)	C11—C12—C13—C14	0.9 (14)
C10—N2—C6—C7	0.7 (16)	C12—C13—C14—C15	−1.0 (14)
N2—C6—C7—C8	−0.5 (17)	C11—N3—C15—C14	0.1 (13)
C6—C7—C8—C9	0.0 (16)	C13—C14—C15—N3	0.5 (14)

### Hydrogen-bond geometry (Å, °)

**Table:**

*D*—H···*A*	*D*—H	H···*A*	*D*···*A*	*D*—H···*A*
N1—H1···Br9	0.88	2.35	3.202 (9)	163
N2—H2···Br9	0.88	2.59	3.292 (8)	137
N3—H3···Br9	0.88	2.52	3.279 (7)	146
